# Newborn’s first bath: any preferred timing? A pilot study from Lebanon

**DOI:** 10.1186/s13104-020-05282-0

**Published:** 2020-09-14

**Authors:** Joelle Mardini, Clara Rahme, Odette Matar, Sophia Abou Khalil, Souheil Hallit, Marie-Claude Fadous Khalife

**Affiliations:** 1 Faculty of Medicine and Medical Sciences, Holy Spirit University of Kaslik (USEK), Jounieh, Lebanon; 2 Pediatrics and Neonatology Department, Notre Dame Des Secours University Hospital, Byblos, Lebanon; 3 Research Department, Psychiatric Hospital of the Cross, Jal Eddib, Lebanon; 4 Gynecology and Obstetrics Department, Notre Dame Des Secours University Hospital, Byblos, Lebanon; 5 INSPECT-LB: Institut National de Santé Publique, Epidémiologie Clinique Et Toxicologie-Liban, Beirut, Lebanon

**Keywords:** Neonates, Bathing time, Incubation time, 24-h status, Vernix caseosa

## Abstract

**Objective:**

To try to find the most appropriate time for the newborn’s first bath. This prospective randomized study was conducted in one hospital (July–September 2017).

**Results:**

A higher percentage of newborns who had a skin-to-skin contact with their mothers had their bath at 24 h vs 2 h after birth (65.2% vs 33.3%; p = 0.01). A higher percentage of mothers who helped in their baby’s bath had their baby’s bath at 24 h vs 2 h (65.2% vs 5.9%; p < 0.001) and vs 6 h (65.2% vs 15.7%; p < 0.001) respectively. A higher mean incubation time was seen between newborns who had their bath at 2 h (2.10 vs 1.78; p = 0.002) and 6 h (2.18 vs 1.78; p = 0.003) compared to those who had their bath at 24 h respectively. A higher percentage of newborns who took their first bath 24 h after birth were calm compared to crying vigorously (38.6% vs 9.1%; p = 0.04). Delaying newborn first bath until 24 h of life was associated with benefits (reducing hypothermia and vigorous crying, benefit from the vernix caseosa on the skin and adequate time of skin-to-skin contact and mother participation in her child’s bathing.

## Introduction

Bathing of the newborn is a part of routine care in hospital nurseries. The aim of the first bath is to remove undesired fluids as blood and meconium on the newborn’s body and to provide hydration to the stratum corneum of the newborn’s skin in order to maintain skin integrity, barrier function property and body temperature [1]. There is an ongoing debate about when the first bath should be given. The World Health Organization (WHO) reports that newborns should not be given a bath in the first 24 h but to wait until their vital signs become stable, especially that this will leave residual vernix caseosa intact allowing it to wear off with normal care and handling [2]. If this is not possible for cultural reasons, bathing should be delayed at least 6 h after birth to allow the neonate to pass into extra-uterine life, thus enabling the bonding of the mother to the newborn [2]. However, the Association of Women’s Health, Obstetric and Neonatal Nurses (AWHONN) recommends giving first bath when cardio-respiratory stability has been achieved, which means to wait up to 2 h after delivery [3]; whereas the National Institute for Health and Clinical Excellence (NICE) says that bathing and other treatments should be initiated no sooner than 1 h after birth, so that maternal-infant contact is not interrupted [4].

One of the many benefits of delaying first bath is waiting for the newborn’s temperature to stabilize around 36.8 degrees Celsius or higher in order to prevent the risk of hypothermia, which is higher in the first hour after birth [5]. A second benefit is allowing to keep intact the vernix caseosa which is a protective fetal film that acts as a chemical and mechanical barrier in utero, with the thickest coating accumulating between 36 and 38 weeks of gestation [6]. The benefits of leaving this coating is protection from infection, skin cleansing and moisturizing and protection from the activity of host defense proteins important for innate immunity [6]. Another idea is that bathing has an influence on skin colonization with microorganisms because it is thought that the skin of the fetus is usually sterile unless there was a premature rupture of membranes [6]. After delivery, a variety of microorganisms will colonize the skin and this is affected by the type of delivery: vaginal or cesarean section. In vaginal delivery, the skin will contain bacteria resembling the mother’s vaginal flora, whereas in cesarean section it will reflect the skin flora of the mother [7].

A very important factor after childbirth is the skin-to-skin contact where the newborn baby is placed naked on the mother's bare chest at birth or shortly afterwards [8, 9]. Evidence supporting the practice of post-birth skin-to-skin contact (SSC) is strong, suggesting multiple benefits for both mother and child. Advantages for the mother include early elimination of the placenta, reduced bleeding, improved self-efficacy of breastfeeding, and decreased maternal stress [9]. Advantages for the infant include a reduction in the negative consequences of “stress of birth”, more appropriate thermoregulation, persisted even in the first days and less crying [9]. Another factor is incubator time defined as the amount of time in the incubator needed to achieve thermal stability [10]. In fact, the first bath should be postponed until the newborn is thermally stable because bathing is associated with a significant loss of heat [10]. The objective of our study is to try to find the most appropriate time for the newborn’s first bath, and conclude if it is compatible with the WHO recommendations.

## Main text

### Methods

#### Study design

**Fig. 1:**
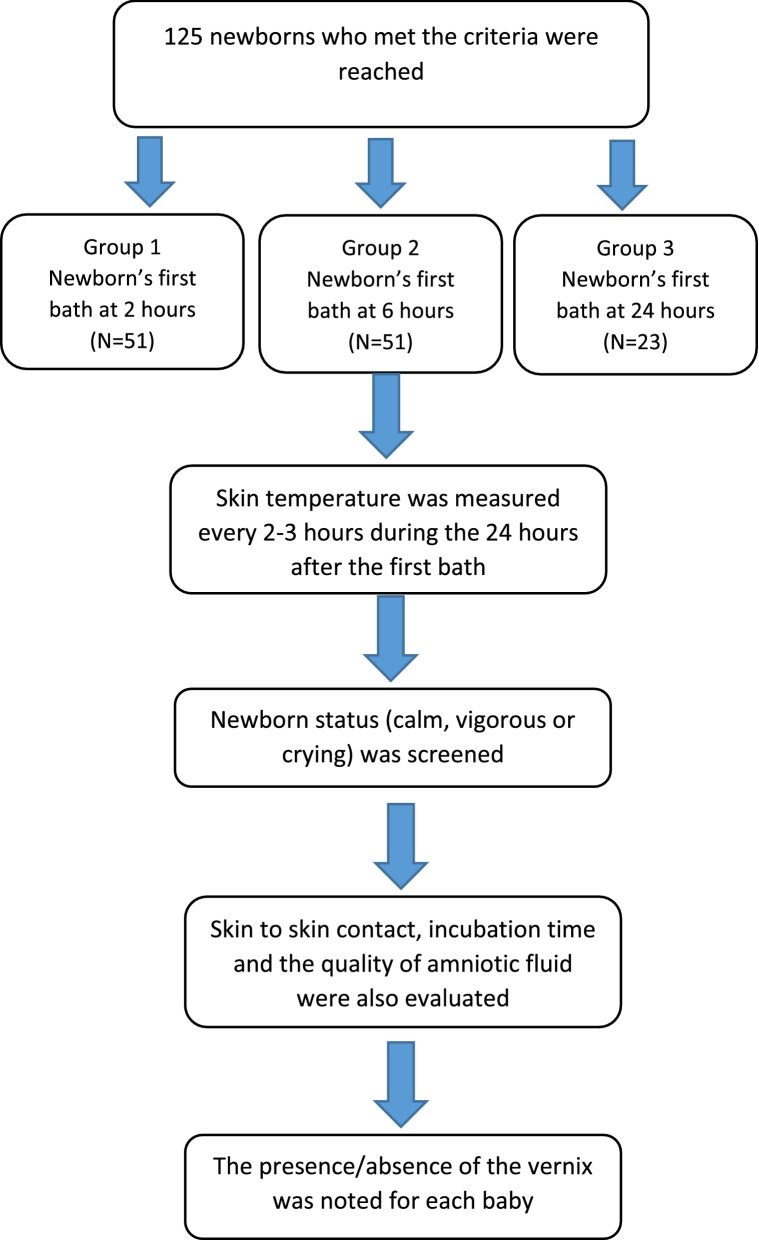
Flow chart summarizing the study design

This pilot study, part of the evaluation of professional practices (EPP; quality improvement project), was prospective, conducted in the maternity department of the Notre-Dame des Secours University Hospital Center from July until September 2017 after approval of the hospital’s ethics committee. Newborns were divided randomly by dice roll into groups: Group 1 when getting number 1 or 2 on the dice, group 2 when getting number 3 or 4 and group 3 when getting number 5 or 6. Group 1 included newborns taking their first bath at 2 h of age, Groups 2 and 3 were formed by newborns taking their first bath at 6 and 24 h of age respectively (Fig. 1). Babies whose mothers requested to bath them earlier than 2 h were excluded from the study. In the absence of similar studies tackling the same outcomes, this study was considered as a pilot one, with no minimal sample size calculated. A total of 125 neonates was enrolled at the end of the data collection. There were no other inclusion/exclusion criteria.

#### Questionnaire

The variables collected were maternal age, mother’s level of education, occupation, age at time of delivery, parity, gestational age, and bathing assistance (mother’s choice to assist with her newborn’s first bath or not). Other variables evaluated any possible association between delaying first bath and age of first skin-to-skin contact (putting the naked baby at the mother’s bare chest directly after primary care has been given), incubation time (time necessary to achieve thermal stability) and newborn status (vigorous crying or calm). All these variables were assessed while taking into consideration the mother’s need and the health care team availability.

The quality of amniotic fluid was noted in every baby and the presence of vernix caseosa on the skin of each baby was evaluated at 2 h, 6 h, and 24 h of age and then noted in each baby’s file as positive (presence of vernix) or negative (absence of vernix). After the first bath, skin temperature was taken every 2 to 3 h for 24 h. A subjective assessment of the general state of the baby was done by two experienced midwives to classify each baby as being calm, sleepy, or having vigorous screaming.

### Statistical analysis

The statistical analysis of the results was made via SPSS version 22. Frequencies/percentages as well as means and standard deviations were used to describe categorical and continuous variables respectively. The sample did not have a normal distribution; therefore, non-parametric tests were used for the bivariate analysis. The Chi-square test was used to compare between categorical variables; the Fisher’s exact test was used when the number of cells was less than 5. The Kruskal–Wallis test was used to compare 3 or more means. P < 0.05 was considered significant.

### Results

Fifty-one children took their bath at 2 h, whereas 51 and 23 children took their bath at 6 and 24 h respectively.

#### Bivariate analysis of factors associated with the newborn’s bath timing

A significant association was seen between the mother-baby skin-to-skin contact and the newborn’s bath timing for the whole trend and between newborns who had their bath at 2 h and 24 h; a significantly higher percentage of newborns who had their bath 2 h after birth did not have a skin-to-skin contact with their mothers directly after birth (after first care was done). (65.2% vs 33.3%; p = 0.01). A significant association was seen between the mother’s assistance with newborn’s bath for the whole trend, and between newborns who had their bath at 2 vs 6 h and 2 vs 24 h respectively. A significantly higher percentage of mothers who helped in their baby’s bath had their baby’s bath at 24 h vs 2 h (65.2% vs 5.9%; p < 0.001) and vs 6 h (65.2% vs 15.7%; p < 0.001) respectively. A significant association was seen between the incubation time and the newborn’s bath timing for the whole trend, and between groups 1 vs 3 and 2 vs 3 respectively. A significantly higher mean incubation time was seen between newborns who had their bath at 2 h (2.10 vs 1.78; p = 0.002) and 6 h (2.18 vs 1.78; p = 0.003) compared to those who had their bath at 24 h respectively (Table 1).

**Table 1:** Bivariate analysis of factors associated with the newborn’s bath timing

Variable	Group 1 (2 h)	Group 2 (6 h)	Group 3 (24 h)	*p between 3 groups*
Age of the mother (in years)				0.543
20–25	7 (13.7%)	8 (15.7%)	4 (17.4%)	
26–30	22 (43.1%)	14 (27.5%)	7 (30.4%)	
> 30	22 (43.1%)	29 (56.9%)	12 (52.2%)	
Education level				0.210
Primary	11 (21.6%)	4 (7.8%)	1 (4.3%)	
Secondary	6 (11.8%)	9 (17.6%)	4 (17.4%)	
University	34 (66.7%)	38 (74.5%)	18 (78.3%)	
Profession				0.571
Unemployed	23 (45.1%)	20 (39.2%)	12 (52.2%)	
Employed	28 (54.9%)	31 (60.8%)	11 (47.8%)	
Delivery method				0.537
Normal	32 (62.7%)	31 (60.8%)	17 (73.9%)	
Cesarean	19 (37.3%)	20 (39.2%)	6 (26.1%)	
Parity				0.899
1	20 (39.2%)	21 (41.2%)	10 (43.5%)	
2	21 (41.2%)	17 (33.3%)	9 (39.1%)	
3 and more	10 (19.6%)	13 (25.5%)	4 (17.4%)	
Skin-to-skin contact mother-baby				0.027
No	34 (66.7%)	25 (49.0%)	8 (34.8%)	
Yes	17 (33.3%)	26 (51.0%)	15 (65.2%)	
Incubation				0.795
No	1 (2.0%)	1 (2.0%)	0 (0%)	
Yes	50 (98.0%)	50 (98.0%)	23 (100%)	
Mother’s assistance with newborn’s bath				< 0.001
No	48 (94.1%)	43 (84.3%)	8 (34.8%)	
Yes	3 (5.9%)	8 (15.7%)	15 (65.2%)	
Vernix caseosa				0.154
No	26 (51.0%)	32 (62.7%)	17 (73.9%)	
Yes	25 (49.0%)	19 (37.3%)	6 (26.1%)	
Number of previous pregnancies	2.24 ± 1.24	2.33 ± 1.54	1.96 ± 0.98	0.747
Duration of the mother-baby skin to skin contact	1.71 ± 0.77	1.52 ± 0.70	1.67 ± 0.62	0.579
Incubation time	2.10 ± 0.36	2.18 ± 0.66	1.78 ± 0.52	0.002

Post hoc analysis: skin to skin contact with the mother (group 1 vs 3: p = 0.01); mother’s assistance with newborn’s bath (group 1 vs 3: p < 0.001 and group 2 vs 3: p < 0.001); incubation time (group 1 vs 3: p = 0.002 and group 2 vs 3: p = 0.003)

A significantly higher percentage of newborns who took their first bath 24 h after birth were calm compared to the other two groups who were crying vigorously (38.6% vs 9.1%; p = 0.04) (Table 2).

**Table 2:** Association between the first bath timing and the newborn status

Variable	Newborn status			*p values*			
	Calm	Somnolent	Crying	*p between 3 groups*	*p 1–2*	*p 1–3*	*p 2–3*
First bath timing				0.07	0.096	0.04	0.690
2 h	35 (34.7%)	6 (75.0%)	10 (62.5%)				
6 h	44 (43.6%)	2 (25.0%)	5 (31.3%)				
24 h	22 (21.8%)	0 (0%)	1 (6.3%)				

p 1–2 = p between groups 1 and 2; p 1–3 = p between groups 1 and 3; p 2–3 = p between groups 2 and 3

### Discussion

The inability of the infant to self-regulate their temperature puts them at risk of many complications, including hypothermia, hypoglycemia and infection, making it important to ensure early stabilization after birth [11].

In this pilot EPP study, a significantly higher percentage of newborns who had their bath 2 h after birth did not have a skin-to-skin contact with their mothers directly after birth (after first care was done). A previous study found that skin-to-skin touch helps in thermoregulation during a newborn transition from intrauterine to extrauterine [10]. Actually, newborn thermoregulation is the main consideration of the timing of the first bath. According to Cheffer and Rannalli, a healthy newborn will develop independent thermoregulation during the transition with temperature stability between 36.4˚C and 37˚C. The transition period was defined as the first minutes after birth and continued up to 24 to 48 h, with major transitions arising within the first 6 to 8 h [10]. To this purpose, an early first bath will lead to the removal of the vernix and the disruption of skin-to-skin contact, which may affect the temperature of the infant and the bonding to his/her mother [12].

In addition, a significantly higher percentage of mothers’ assistance to their baby’s bath was seen in the group of babies bathed at 24 h vs 2 h and vs 6 h respectively. This is in line with a previous research, which claimed that the delayed bathing technique enabled parents to take part in their newborn first bath [13]. Indeed, parents seemed more at ease and open to learning, and some were eager to take part in the bathing. This is an added benefit to demonstrate a healthy bath and have parents engage in an experience that can be overwhelming for first-time parents [13].

Our study showed that the majority of newborns who were bathed at 2 h had to be reheated in the incubator for a longer period than the group of newborns taking their bath at 24 h of life.; They were put in the incubator for 1 h to monitor spO2 and temperature, if temperature went back to neutrality (between 36.5 and 37.5 degrees Celsius the baby was taken out of the incubator). The lowest temperature we had was 36 degrees. Newborns are particularly sensitive to changes in ambient temperature due to their small body mass and relatively large body surface areas [10]. A randomized study trial performed by Bergman et al. found better thermoregulation and cardiorespiratory safety in infants with SSC relative to those cared for in the incubator [14].

Furthermore, a significantly higher percentage of newborns who took their first bath 24 h after birth were calm compared to those who took their bath at 2 h. In reality, the babies who took their bath 24 h after birth profited from the SSC, which gave them comfort and safety for the babies [12].

### Conclusion and clinical implications

In our study, delaying newborn bathing beyond 12 h of life, especially waiting till 24 h of life was found to have several benefits. Beside the reduction of the risk of hypothermia and the need for warming in the incubator, it reduced vigorous crying and allowed the baby to benefit from the protective and moisturizing properties of the vernix caseosa, not to forget the most important benefit of all which was the satisfaction of mothers who were able to assist in their child bath especially when adequate skin to skin contact was provided at birth allowing mother and baby bond, especially that skin-to-skin contact is an important factor that enhances exclusive breastfeeding rates which is a topic that we are always promoting in our maternity department.

## Limitations

Our study had some limitations. First, the type of the study being monocentric could lead to selection bias. Besides, an under or over estimation of a question could be experienced by the mother, leading to an information bias. In addition, the evaluation of the baby’s state during the day and the evaluation of the presence of vernix caseosa were subjective (assessment made by the midwives without a specific score to follow), and data about temperatures and mean age of first skin-to-skin contact were not collected. More than that, the number of health care workers required to follow the bathing schedule as planned was limited, along with the workload of the nursery. Finally, the small number of children enrolled does not allow the extrapolation of the results to the general population. Future studies, considering all these limitations, should be conducted in order to confirm our results.

## Data Availability

There is no public access to all data generated or analyzed during this study to preserve the privacy of the identities of the individuals. The dataset that supports the conclusions is available to the corresponding author upon request.

## Abbreviations

WHO: World Health Organization
AWHONN: Association of Women’s Health, Obstetric and Neonatal Nurses
NICE: National Institute for Health and Clinical Excellence
SSC: Skin-to-skin contact
